# Mechanism of Heparin Acceleration of Tissue Inhibitor of Metalloproteases-1 (TIMP-1) Degradation by the Human Neutrophil Elastase

**DOI:** 10.1371/journal.pone.0021525

**Published:** 2011-06-23

**Authors:** Gabriel L. C. Nunes, Alyne Simões, Fábio H. Dyszy, Claudio S. Shida, Maria A. Juliano, Luiz Juliano, Tarsis F. Gesteira, Helena B. Nader, Gillian Murphy, Alain F. Chaffotte, Michel E. Goldberg, Ivarne L. S. Tersariol, Paulo C. Almeida

**Affiliations:** 1 Centro Interdisciplinar de Investigação Bioquímica, Universidade de Mogi das Cruzes, Mogi das Cruzes, Brazil; 2 Departamento Materiais Dentários, Universidade de São Paulo, São Paulo, Brazil; 3 Grupo de Biofísica Molecular, Universidade de São Paulo, São Carlos, São Paulo, Brazil; 4 Departamento de Biofísica, Universidade Federal de São Paulo, São Paulo, Brazil; 5 Departamento de Bioquímica, Universidade Federal de São Paulo, São Paulo, Brazil; 6 Department of Oncology, University of Cambridge, Cambridge, United Kingdom; 7 Unité de Résonance Magnétique Nucléaire des Biomolécules, Institut Pasteur, Paris, France; 8 Unité de Repliement et Modelisation des Protéines, Institut Pasteur, Paris, France; National Institute for Medical Research, Medical Research Council, London, United Kingdom

## Abstract

Heparin has been shown to regulate human neutrophil elastase (HNE) activity. We have assessed the regulatory effect of heparin on Tissue Inhibitor of Metalloproteases-1 [TIMP-1] hydrolysis by HNE employing the recombinant form of TIMP-1 and correlated FRET-peptides comprising the TIMP-1 cleavage site. Heparin accelerates 2.5-fold TIMP-1 hydrolysis by HNE. The kinetic parameters of this reaction were monitored with the aid of a FRET-peptide substrate that mimics the TIMP-1 cleavage site in pre-steady-state conditionsby using a stopped-flow fluorescence system. The hydrolysis of the FRET-peptide substrate by HNE exhibits a pre-steady-state burst phase followed by a linear, steady-state pseudo-first-order reaction. The HNE acylation step (*k*_2_ = 21±1 s^−1^) was much higher than the HNE deacylation step (*k*_3_ = 0.57±0.05 s^−1^). The presence of heparin induces a dramatic effect in the pre-steady-state behavior of HNE. Heparin induces transient lag phase kinetics in HNE cleavage of the FRET-peptide substrate. The pre-steady-state analysis revealed that heparin affects all steps of the reaction through enhancing the ES complex concentration, increasing *k*_1_ 2.4-fold and reducing *k*_−1_ 3.1-fold. Heparin also promotes a 7.8-fold decrease in the *k*_2_ value, whereas the *k*_3_ value in the presence of heparin was increased 58-fold. These results clearly show that heparin binding accelerates deacylation and slows down acylation. Heparin shifts the HNE pH activity profile to the right, allowing HNE to be active at alkaline pH. Molecular docking and kinetic analysis suggest that heparin induces conformational changes in HNE structure. Here, we are showing for the first time that heparin is able to accelerate the hydrolysis of TIMP-1 by HNE. The degradation of TIMP-1is associated to important physiopathological states involving excessive activation of MMPs.

## Introduction

Human neutrophil elastase (HNE) is a powerful serine proteinase secreted by neutrophils, the first cells recruited to inflammatory sites. HNE is present at concentrations in the milimolar range in the azurophil granules of neutrophils [1]. HNE is a very basic protein with a pI >9.0; eighteen arginine residues are arranged to the surface of the enzyme in a horseshoe-like manner around the active site [2], they strongly interact with heparin-like glycosaminoglycans [3], [4]. HNE depends on serglycin proteoglycans for localization in azurophil granules. Serglycin proteoglycan, the major intracellular proteoglycan of hematopoietic cells, has been related to sorting and packing of granule proteins [5].

Neutrophil elastase/antielastase imbalance is related to uncontrolled proteolytic injury in several chronic inflammatory diseases [6]. It has been shown that heparin is capable of decreasing the inhibitory activity of α1-antitrypsin inhibitor and Mucus Proteinase Inhibitor upon HNE and neutrophil cathepsin G [7]. In sputum sols of patients with bronchiectasis, shed syndecan-1 restricts HNE from α1-antitrypsin, the interaction of HNE with heparan sulfate polysaccharide chain lead to unopposed neutrophil elastase activity, despite overwhelming excess of the physiological antielastase, α1-antitrypsin inhibitor [8]. Interestingly, cellular heparan sulfate proteoglycans (HSPG) can anchor HNE at the cell surface of neutrophils; this interaction preserves the catalytic activity of HNE upon its natural substrates, fibronectin and elastin [9]. HNE binding to HSPG at the neutrophil surface focuses the activity of this potent proteolytic enzyme to the pericellular environment and also preserves its activity by protecting it from inhibition by α1-antitrypsin and SLPI [10].

It has been shown that heparan sulfate proteoglycans syndecan-1 and syndecan-4 maintain the proteolytic balance in acute wound fluid. Syndecan-1 ectodomain protects cathepsin G from inhibition by α1-antichymotrypsin and squamous cell carcinoma antigen 2, and it protects neutrophil elastase from inhibition by α1-proteinase inhibitor. Moreover, the degradation of endogenous heparan sulfate from wound fluids reduces proteolytic activities in the fluid [11]. Syndecan knockout mice show deficits in tissue repair [12]. Taken together, these data show that heparan sulfate proteoglycans are orchestrating the inflammatory response in the process of tissue repair [13].

TIMP-1 is tightly correlated to the maintenance of extracellular matrix (ECM) structure, by acting as inhibitor of MMP-2 and MMP-9. Extracellular matrix degradation is observed in several physiopathological conditions, such as tumor cell invasion, arthritis, metastasis and inflammatory processes [14]. It has been shown that TIMP-1 and MMP-9 activities can be regulated by HNE activity. HNE preferentially inactivates TIMP-1 in the pro-MMP-9.TIMP-1 complex and renders pro-MMP-9 activatable by MMP-3 [15]. HNE was shown to be able to inactivate TIMP-1 through the cleavage of a single peptide bond Val^69^-Cys^70^ [16]. The activation of MMP-9 and the TIMP-1 inactivation by HNE have important physiopathological role in cystic fibrosis lung disease [17], intracranial hemorrhage [18], abdominal aortic aneurysm [19] and bone resorption [20].

In this study, we have investigated the influence of heparin upon HNE activity in the inactivation on TIMP-1. A combination of SDS-PAGE, FRET-peptide substrate assays in stopped-flow fluorescence kinetic measurements and molecular docking, was used to characterize the interaction of HNE with heparin. Here, we are showing for the first time that heparin is able to accelerate the hydrolysis of TIMP-1 by HNE. The excessive degradation of TIMP-1is associated to important physiopathological states involving activation of MMP-9.

## Materials and Methods

### Materials

HNE (EC 3.4.21.37) was purchased from Calbiochem/Novabiochem, (LaJolla, USA). Fluorogenic substrate MeOSuc-AAPV-MCA, irreversible inhibitor of HNE MeO-Suc-AAPV-CH2Cl and serine proteinase inhibitor PMSF were purchased from Sigma-Aldrich (USA). Human recombinant TIMP-1 was prepared as described previously [21]. Heparin 14 kDa was purchased from Calbiochem (La Jolla, USA). The Fluorescence Resonance Energy Transfer (FRET)-peptide containing *ortho*-aminobenzoic acid (Abz) as donor group and N-(2,4-dinitrophenyl) ethylenediamine (EDDnp) as acceptor group, Abz-AMESVMGYFHRSQ-EDDnp was synthesized in solid phase chemistry as described below.

### Chemical Synthesis of FRET-peptide

The Fluorescence Resonance Energy Transfer (FRET)-peptide substrate containing *ortho*-aminobenzoic acid (Abz) as donor group and N-(2,4-dinitrophenyl) ethylenediamine (EDDnp) as acceptor group, Abz-AMESVMGYFHRSQ-EDDnp was synthesized in solid phase chemistry as described previously [22]. An automated bench top simultaneous multiple solid-phase peptide synthesizer (PSSM 8 system from Shimadzu) was used for solid-phase synthesis of the FRET-peptide substrate by Fmoc-procedure. The final deprotected peptides were purified by semi preparative HPLC using an Econosil C-18 column (10 µm, 22.5×250 mm) and a two solvent system: (A) TFA/H_2_O (1∶1000) and (B) TFA/acetonitrile (CAN)/H_2_O (1∶90∶10). The column was eluted at a flow rate of 5 mL/min with a 10 (or 30) to 50 (or 60)% gradient of solvent B over 30 or 45 min. Analytical HPLC was performed using a binary HPLC system from Shimadzu with SPD-10AV Shimadzu UV-*vis* detector and a Shimadzu RF-535 fluorescence detector, coupled to an Ultrasphere C-18 column (5 µm, 4.6×150 mm) which was eluted with solvent systems A and B at a flow rate of 1 mL/min and 10 to 80% gradient of B over 20 min. The elution of peptides in HPLC column was monitored by absorbance at 220 nm and by fluorescence emission at 420 nm following excitation at 320 nm. The molecular weight and purity of synthesized peptides were checked by MALDI-TOF mass spectrometry (TofSpec-E, Micromass) and/or peptide sequencing using a protein sequencer PPSQ-23 (Shimadzu Tokyo, Japan).

### Kinetic Studies

HNE endopeptidase activity was monitored fluorometrically using either the FRET substrate Abz-AMESVMGYFHRSQ-EDDnp or the fluorogenic substrate MeOSuc-AAPV-MCA. The fluorescence intensity was monitored on a thermostatic Hitachi F-2500 spectrofluorimeter. The steady-state kinetic assays with fluorogenic substrates were performed in 10 mM Hepes (pH 7.4) buffer containing 140 mM NaCl and 0,05% Triton X-100 at 37°C. The concentration of active HNE was determined by titration with the irreversible chloromethylketone inhibitor MeO-Suc-AAPV-CH2Cl. All reactions were done in 1×1 cm cross section quartz cuvette. For MeOSuc-AAPV-MCA (0.02–1.00 mM) substrate assays, the excitation and emission wavelengths were set at 380 and 460 nm, respectively. For the FRET-peptide substrate (0.2–10 µM), the assay was conducted at 420 nm using an excitation wavelength of 320 nm. The kinetic parameters were determined by measuring the initial rate of hydrolysis at various substrate concentrations in presence and or absence of heparin. The fluorescence of 7-amino-4-methylcoumarin and *ortho*-aminobenzoic acid were determined for the calculation of precise rateconstants. In order to calculate the concentration of the released product calibration curves of fluorescence versus concentration were constructed. The data obtained were analyzed by nonlinear regression using the program GraFit 3.01 (Erithacus Software Ltd.). The data were analyzed in steady-state kinetic system and the values for the constants were determined by using nonlinear regression to the hyperbolic tight-binding equations as previously suggested [4]. The peptide bonds cleaved in enzymatic reactions were determined by matrix-assisted laser-desorption ionization-time-of-flight mass spectrometry (TofSpec-E; Micromass, Manchester, U.K.).

### Stopped-Flow Kinetic Analysis

Stopped-Flow fluorescence kinetic measurements were carried out at 25°C with an SFM300 mixing device from Bio-Logic (Pont de Claix, France) equipped with two large (10 ml) syringes injecting through the first mixer (*S1*) 25 µl of substrate and (*S2*) 365 µl of reaction buffer 10 mM Tris-HCl (pH 7.4) containing 100 mM NaCl in presence or absence of heparin and a small syringe (*S3;* 2.5 ml) injectingthrough the second mixer 10 µl of HNE diluted in the same buffer as that of the substrate. The injection time was 45 ms, (dead time of 3.1 ms). The signal recording was triggered at the end of injection. The mixing device, equipped with an Fc15 flow cell (1.5 mm×1.5 mm cross section), was combined with the optical bench and detection module of Bio-Logic. The incident wavelength was set at 320 nm from a monochromator with a bandpass of 8 nm. The emitted light was collected by a photomultiplier through a 350 nm high pass filter for Abz fluorescence measurements. The signal was electronically processed in the Bio-Logic amplifier, and finally converted into data files by means of the Bio-Kine software package from Bio-Logic. In the experiments in the presence of heparin the final concentrations were 3.8 µM FRET-peptide Abz-AMESVMGYFHRSQ-EDDnp, 50 µM heparin and 12.7 nM HNE. All the solutions were filtered and degassed immediately before they were used.

### Stopped-Flow Data analysis

The kinetic data were processed using DynaFit IV [23]. Regression analysis in DynaFit is performed by Reich's variation of the Levenberg–Marquardt least-squares fitting algorithm. Convergence criteria are multiple in DynaFit: The Marquardt parameter for individual parameters should be met first. This was typically met within 100 iterations and 50 subiterations. Standard errors of the individual parameters were computed from the square roots of diagonal elements of the final variance–covariance matrix. The protocol that gave individual rate constants and Michaelis–Menten parameters were calculated from them, in agreement with experimentally determined individual rate constants and experimentally determined Michaelis–Menten parameters.

The fitting started with a three-step consecutive mechanism as previously described [24]:
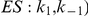


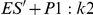


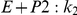



From which the following differential equations were derived:
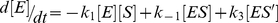
(Eq. 1)

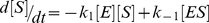
(Eq. 2)

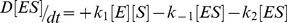
(Eq. 3)

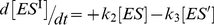
(Eq. 4)

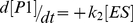
(Eq. 5)

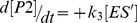
(Eq. 6)


The initial guesses were based on experimentally determined values of *k*_cat_, which were used for the estimation of *k*_2_; the concentration of enzyme and substrate were determined independently. In a second round, *k*_1_, *k*_−1_, *k*_2_ and *k*_3_ were optimized by fitting for individual runs of 6,000 and 4,000 fluorescence time data pairs, covering a total period of 6 and 4 sec, corresponding to experiments in absence and presence of heparin, respectively, on the same three-step mechanism mentioned above [25]. From the three-step reaction scheme, 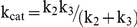, 
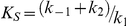
, 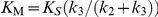 and 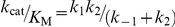 were calculated values [24]. The same kinetic constants calculation steps described were used for the experiments in presence of large molar excess of 50 µM heparin.

The kinetic events corresponding to heparin, HNE and substrate reactions can be described by the model represented in Scheme I.

Scheme I
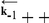


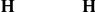


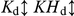


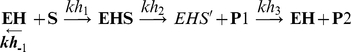



### The influence of heparin upon HNE proteolysis of TIMP-1

TIMP-1 (3.1 µM) was incubated with 5.5 nM HNE in the absence or in the presence 50 µM heparin for various periods of time. The enzymatic reaction was performed in 100 mM Tris-HCl buffer (pH 7.4) containing 100 mM NaCl and 0,05% Triton X-100 at 37°C. Aliquots of both reaction mixtures were collected at 20, 40, 60, 90 and 120 minutes and the reaction was stopped by addition of PMSF to 0.1 mM final concentration in SDS-PAGE sample buffer consisting of 200 mM Tris-HCl (pH 7.0) 4% SDS, 10% 2-mercaptoethanol, 20% glycerol, 0.025% bromphenol blue (1∶1 v/v) and finally boiled for 5 min. Samples were submitted to 12.5% SDS-PAGE and the products of TIMP-1 hydrolysis were visualized by silver staining. TIMP-1 fragments were detected by scanning densitometry.

### HNE pH Activity Profile

For the determination of pH activity profiles, the kinetics of FRET-peptide substrate hydrolysis were performed in absence or in presence of 50 µM heparin at 37°C in four-component buffer system of constant ionic strength, consisting of 25 mM glycine, 25 mM acetic acid, 25 mM Mes and 75 mM Tris, containing 140 mM NaCl and 0.05% (v/v) Triton X-100, the pH of buffers were adjusted using HCl or NaOH diluted solutions. The substrate concentrations were kept 20-fold below the *K*_M_ values. The progress of the reaction was continuously monitored by the fluorescence of the released product. The initial rates were determined, and the *k*_cat_/*K_M_* values were obtained by dividing the initial rates by enzyme and substrate concentrations. It is important to mention that the enzyme was stable at pH range studied, and the pH values did not affect the ionic form of substrate. The pH activity profiles data were fitted according to Equation 7 by using non-linear regression software system (GraFit version 3.0, Erithacus Software Ltd) as follows:
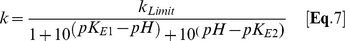



Equation 7 fits data when the pH-activity profile depends upon two ionizing groups in a bell-shaped curve and the activities at low and high pHs are zero;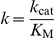; *k_Limit_*stands for the pH-independent maximum rate constant, p*K*_E1_and p*K*_E2_are the dissociation constants of a catalytically competent base and acid respectively.

### Molecular Docking

PatchDock [26] was used to dock heparin to HNE complexed with 1/2SLPI (Protein Data Bank ID 2Z7F). PatchDock is a fast geometry-based docking algorithm that optimizes shape complementarily, used for an initial analysis of surface and atomic contact variables, with no constraints used to define the initial binding site. All docking simulations were performed using heparin di- and octasaccharides. The octasaccharide has been modeled with the iduronic acid at the non-reducing end in a ^1^H_2_ conformation and the central and terminal iduronic acids in the ^1^C_4_ conformation, and the disaccharide (IdoA,2S (1→4)Glc*N*S,6S) was extracted from a heparin fragment (Protein Data Bank code 1HPN) [27], [28]. Different docking simulations with iduronic acid in the ^1^C_4_ chair conformation and the glucosamine in the ^1^C_4_ chair conformation were performed in order to compare the docking results with those obtained for a similar conformation of the octasaccharide. Oligosaccharide structures were energy-minimized after addition of hydrogen atoms to optimize the orientation of rotatable groups and the surface area, contact energy, and binding score were extracted from PatchDock. AutoDock4.0 was used as a grid based docking procedure [29], defining a grid of 0.46 Å and distance dependent dielectric constant of 4.0 for the binding energy calculations. The genetic algorithm with local search options (GA-LS) as implemented in AutoDock was used to dock the flexible heparin fragments, with 4500 search runs using an initial population of 200 conformations. Hydroxyl groups were kept fixed in order to maintain a maximum of 32 rotatable bonds, and the grid box and constant grid spacing of 0.46 Å around each heparin fragment binding were obtained from PatchDock with respect to HNE.

The FRET-peptide docking was built using PRODRG2 [30], with Gasteiger charges [31] calculated using Autodock tools [http://www.scripps.edu/~sanner/python/adt/]. Lamarckian genetic algorithm, with 2.5×10^8^ energy evaluations (high) and 200 generations with step sizes of 0.2 for translation and 5.0 for quaternion and torsion, respectively, was employed. The best-docked conformers with lowest free energies (−14 kcal/mol) conformations were taken for further analysis.

## Results

### Effect of Heparin on the Proteolytic Processing of TIMP-1 by HNE

**Figure 1:**
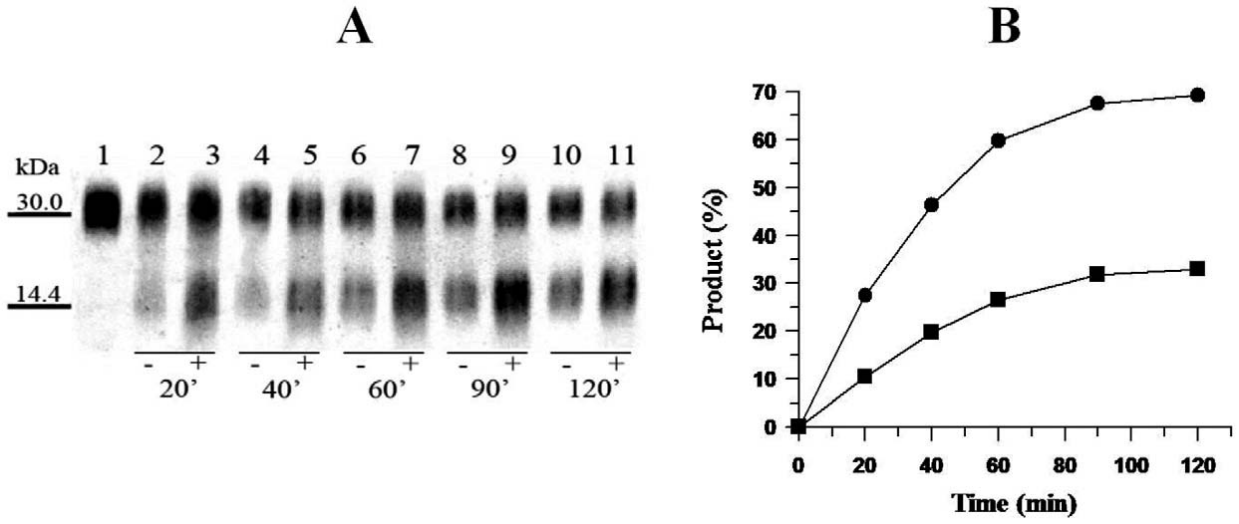
Heparin up-regulates TIMP-1 proteolysis by HNE. *A*, TIMP-1 (220 *µ*g/ml) was allowed to react with 0.55 *µ*g/ml HNE at 37°C in absence (lanes 1, 2, 4, 6, 8 and 10) or in presence (lanes 3, 5, 7, 9 and 11) of 50 µM heparin. The reaction was performed in 100 mM Tris-HCL, pH 7.4, containing 100 mM NaCl, and 0,05% Triton X-100. Aliquots from both samples were collected at 20, 40, 60, 90 and 120 minutes and the reaction was stopped by inactivation of HNE with PMSF. TIMP-1 fragments were analyzed by SDS-PAGE under reducing conditions and visualized by silver staining. *B*, the extent of TIMP-1 cleavage in the presence (•) or in the absence of heparin (▪) is plotted as a function of time of reaction with HNE after gel densitometry.

It has been shown that HNE is able to cleave TIMP-1 at the Val^69^-Cys^70^ bond [16]. Since heparin and HSPG can modulate the activity of HNE, weexamined whether heparin modified the proteolysis of TIMP-1 by HNE. Figure 1 shows a SDS-PAGE image analyzing the proteolytic processing of TIMP-1 by HNE in the absence or in the presence of 50 µM heparin. The efficiency of TIMP-1 cleavage by HNE in the presence or in the absence of heparin was indicated by the appearance of the 14 kDa and 16 kDa fragments [16]. Scanning densitometry of time course experiments indicated that heparin increased 2.5-fold the initial rate of TIMP-1 14 kDa and 16 kDa fragments released by HNE (Fig. 1B). This result is very interesting and it was not expected since heparin [3], [4] and other natural polyanionic polymers such as DNA [32] and alginate [33] have been described in the literature as inhibitors of HNE.

### Effect of Heparin upon the HNE Endopeptidase Activity

In order to better study the mechanism of heparin action on the hydrolysis of TIMP-1 by HNE, we synthesized a FRET-peptide Abz-AMESVMGYFHRSQ-EDDnp that mimics the primary sequence of TIMP-1 between residues 65-76 [16]. The HPLC and Maldi-TOF mass spectrometry analysis showed that Val-Met is the only peptide bond cleaved by HNE on this substrate. This FRET-peptide substrate covers the HNE subsites from S_5_ to S'_5_ according to the Schechter & Berger nomenclature [34], which are the main substrate binding sites in HNE [2]. The presence of heparin did not change the pattern of cleavage of this peptide by HNE. The specificity observed in this cleavage is in agreement with that described for HNE in which the subsite *S_1_* is preferentially occupied by short hydrophobic residues such as Val, Ala, Ile and Met [2], [35]. Also, it is important to mention that no interaction between FRET-peptide substrate with heparin was detected by studying the intrinsic fluorescence of FRET-peptide in function of heparin concentration (data not shown).

**Figure 2:**
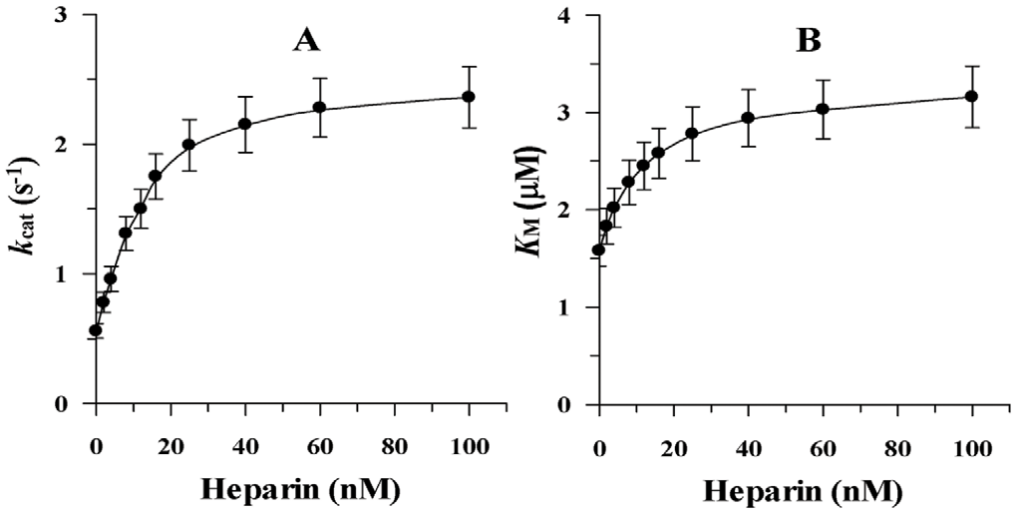
The effect of heparin on steady-state FRET-peptide hydrolysis by HNE. The influence of heparin concentration upon steady-state HNE kinetics parameters *k*_cat_ [A] and *K*_M_ [B] was determined spectrofluometricallyas described under “Experimental Procedures”. The fluorescence increase of FRET-peptide hydrolysis (0.2 - 10 µM) by 12.6 nM HNE were performed in 10 mM Hepes, pH 7.4, containing 140 mM NaCl and 0,05% Triton X-100 at 37°C.

The binding of heparin to HNE perturbs its catalytic activity upon FRET-peptide substrate (Fig. 2). The efficiency of the system for the hydrolysis of FRET-peptide can be altered by changing either *K*_M_ value (α parameter) or *k*_cat_ value (β parameter). Figure 2 shows that the presence of heparin results in a large increase in *k*_cat_ value (β = 4.6±0.2) but also a marked decrease of the affinity of the enzyme for the FRET-peptide substrate as reflected by the strong increase of the K_M_ value (α = 2.0±0.1). The catalytic efficiency (β/α ratio) for this substrate in the presence of heparin thus increased 2.5-fold. Likewise, at high substrate concentration, the dissociation constant of HNE for heparin is increased 2.0-fold. It was observed that heparin binds tightly to free HNE (E) and to the enzyme-substrate complex (ES) with dissociation constants *K*_H_ and α*K*_H_ of 6±1 nM, and 14±1 nM respectively. It was observed that heparin regulates HNE endopeptidase activity upon the substrate Abz-AMESVMGYFHRSQ-EDDnp by a tight-binding hyperbolic mixed type reaction as depicted in Scheme I.

Curiously, heparin showed a different pattern of HNE inhibition when assayed with the fluorogenic substrate MeOSuc-AAPV-MCA. The effects of heparin upon the HNE hydrolysis of this substrate can be described by a tight-binding hyperbolic mixed-type inhibition model. Indeed, the data could be well fitted to an equation that describes this inhibition behavior [4] by using non linear regression, provided the values for the constants shown in Table 1. The presence of heparin in the HNE kinetic assays also results in 2.5 fold increase of the *K*_M_ value for the hydrolysis of the substrate MeOSuc-AAPV-MCA. On the other hand, heparin induced a small decrease in the catalytic constant (*k*_cat_) of HNE for the hydrolysis of this substrate. Interestingly, Spencer at al. [4] have shown that inhibition of HNE hydrolysis of the substrate MeOSuc-AAA-*p*NA by various heparin compounds follows a tight-binding, partial competitive mechanism (α = 3.4±0.3, β = 0.96±0.05). The conjunction of these and our data strongly suggest that the effect of heparin on HNE activity is very dependent on the substrate structure.

**Table 1:** Steady-state kinetic parameters for hydrolysis of fluorogenic substrates by human neutrophil elastase in the presence of heparin.

Substrate	*K*_M_(µM)	*k*_cat_(s^−1^)	*k*_cat_/*K*_M_(mM^−1^s^−1^)	α(*K*_M_^ap^/*K*_M_)	β(*k*_cat_^ap^/*k*_cat_)
*FRET-peptide*					
Control	1.6±0.1	0.56±0.06	350±40		
Heparin	3.1±0.3	2.50±0.20	810±60	2.0	4.46
*MeOSuc-AAPV-MCA*					
Control	138±16	16.8±1.2	106±9		
Heparin	389±40	10.4±0.9	27±4	2.8	0.62
*MeOSuc-AAA-MCA* a					
Control	1610±30	0.36±0.02	0.228±0.003		
Heparin	5940±12	0.36±0.02	0.061±0.001	3.5	0.96

a Reference [4].

Although the three-step mechanism for HNE-catalyzed hydrolysis of peptides is widely accepted [36], the individual rate constants that describe the steps have not been determined in the studies of HNE's inhibition. Since
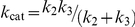
and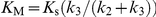 are composites parameters of the rate constants, the Michaelis-Menten kinetic parameters *k*_cat_ and *K*_M,_ determined by steady-state analysis do not give a detailed picture about the kinetic mechanism of HLE inhibition by heparin [24]. Clearly, the only way to get an accurate picture of HNE inhibition by heparin is through the determination of heparin effect upon the mechanistic kinetic parameters *k*_1_, *k*_−1_, *k*_2_ *and k*_3_.

### Pre Steady-State Analysis of the FRET-Peptide Substrate Hydrolysis by HNE

**Figure 3:**
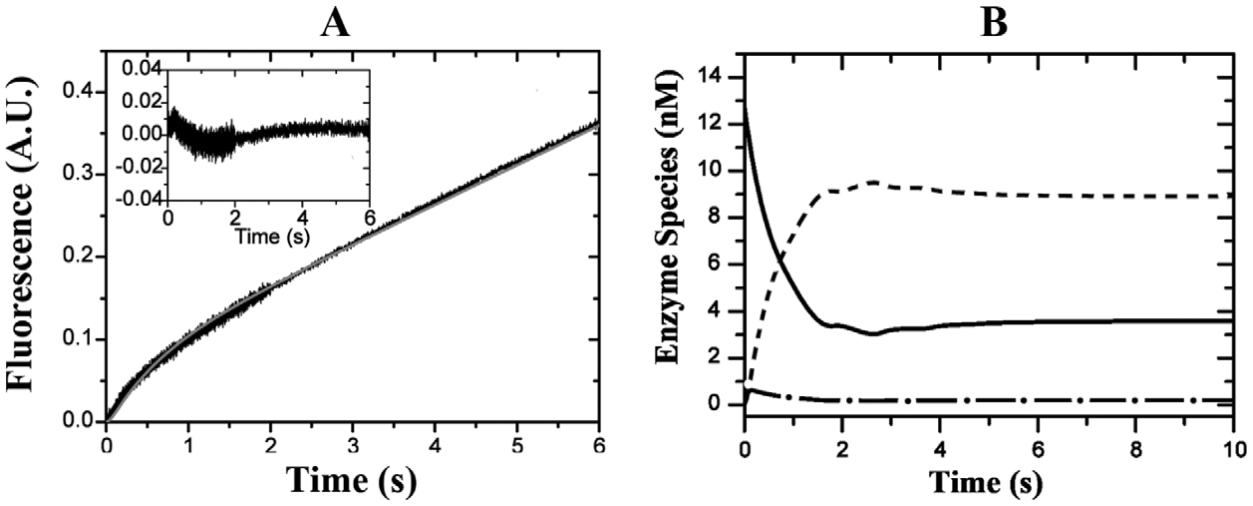
Stopped-flow time-tracesofFRET-peptidehydrolysis by HNE. *A* **,** Stopped-flow fluorescence kinetic recording of 3.8 µMFRET-peptide hydrolysis by 12.6 nM HNEat 25°C in 10 mM Tris-HCl buffer, pH 7.4, containing 100 mM NaCl. The progress of the reaction was monitored by the fluorescence increase of the released product recorded on 2 adjacent time regions with distinct sampling periods: 0.5 ms from 0 to 2 s, 2 ms from 2 to 6 s. Gray solid line represents the best fit obtained from the mechanism depicted in Scheme I in the absence of heparin with the aid of DynaFit IV® software (see Experimental Procedures). The insert graphic represents the associate residual errors from the best fit curve with experimental data. *B*, the HNE species as a function of time reaction: free enzyme, E (**–**); complex enzyme-substrate, ES **(–** •**–**) and acyl-enzyme, ES' (**- - -**).

The kinetics of the FRET-peptide substratehydrolysis by HNE was also conducted in a stopped-flow fluorescence apparatus in the absence or in the presence of 50 µM heparin. Figure 3A shows that in the absence of heparin, the cleavage of the FRET-peptide substrate Abz-AMESVMGYFHRSQ-EDDnp by HNE exhibited an initial burst of product formation, strongly suggesting the existence of a rate-limiting step after acyl-enzyme formation [24],[37]. The observation of such a burst under pre-steady conditions is typical of a rate-limiting deacylation (*k*_3_) in amide hydrolysis [36].

In order to determine the values for the kinetic constants *k*_1_, *k*_-1_, *k*_2_ *and k*_3_ of the FRET-peptide hydrolysis by HNE, the kinetic data from progress curves were processed using DynaFit IV software as previously described. Table 2 shows that the hydrolysis of FRET-peptide by HNE can be satisfactorily described by a three-step kinetic mechanism, where *K*_S_ = (*k*_-1_+*k*_2_)*/k*_1_ stands for the formation of HNE-FRET-substrate complex, *k*_2_ stands for the HNE acylation step and *k*_3_ stands for the HNE deacylation step [24]. As expected, the value of *k*_2_ (21±1 s^−1^) was much higher than the value of *k*_3_ (0.57±0.05 s^−1^) and the *k*_cat_ constant is practically governed by *k*_3_, these values agree with the observation of burst of product formation in the initial step of HNE reaction. Because *k*_2_ (21±1 s^−1^) is much higher than *k*_−1_ (0.25±0.02 s^−1^), the hydrolysis of FRET-peptide substrate by HNE is a diffusion controlled process; the constant of specificity *k*_cat_/*K*_M_ is governed by the association rate constant *k*_1_ (0.35±0.03 µM^−1^.s^−1^). Moreover, as *k*_2_ is largely higher than *k*_−1,_ *K*_M_ (1.6±0.01 µM) cannot be considered a true dissociation constant. These results show that the HNE mainly exists as an acyl-enzyme (71%) in the steady-state during the hydrolysis of this FRET-peptide (Fig. 3B).

**Table 2:** Effect of heparin on pre-steady-state kinetic parameters for hydrolysis of FRET-peptide substrate by human neutrophil elastase.

Conditions	*k*_1_.10^6^(M^−1^s^−1^)	*k*_−1_(s^−1^)	*k*_2_(s^−1^)	*k*_3_(s^−1^)	*K*_M_ ^a^(µM)	*k*_cat_ ^b^(s^−1^)
FRET-peptide						
Control	0.35±0.03	0.25±0.02	21±1	0.57±0.05	1.6±0.1	0.56±0.06
Heparin	0.82±0.06	0.081±0.005	2.7±0.2	33±2	3.1±0.3	2.5±0.2

a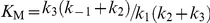 and

^b^ 
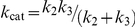
 [36].

**Figure 4:**
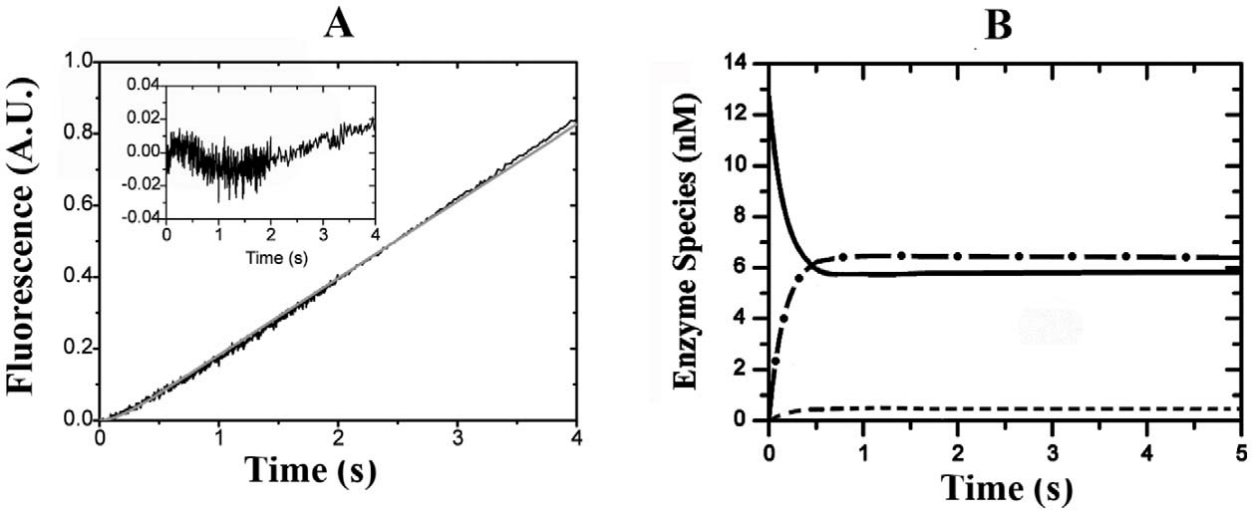
Stopped-flow time-traces of FRET-peptidehydrolysis by HNE in the presence of heparin. *A*, the stopped-Flow fluorescence kinetic recording of 3.8 µMFRET-peptide hydrolysis by 12.6 nM HNEperformed at 25°C in 10 mM Tris-HCl buffer, pH 7.4, containing 100 mM NaCl supplemented with 50 µM heparin. The progress of the reaction was monitored by the fluorescence increase of the released product recorded on two adjacent time regions with distinct sampling periods: 0.5 ms from 0 to 2 s, 2 ms from 2 to 4 s. Gray solid line represents the best fit deduced from the mechanism depicted in Scheme I in the presence of heparin using DynaFit IV® Software [see Experimental Procedures]. The insert graphic represents the associate residual errors from the best fit curve with experimental data. *B*, the HNE species in function of time reaction in presence of heparin: complex enzyme-heparin, EH (**–**); complex enzyme-substrate-heparin, ESH **(–** •**–**) and complex acyl-enzyme-heparin, ES'H (**- - -**).

We were interested in whether heparin could influence the pre-steady-state behavior of the HNE. Interestingly, the presence of heparin induces a dramatic effect in the pre-steady-state behavior of the enzyme. As shown in Figure 4A, heparin induces a transient lag phase in HNE cleavage of FRET-peptide substrate (Fig. 4A). The presence of heparin thus resulted in the loss of the burst in HNE cleavage of FRET-peptide substrate,indicating that deacylation was no longer rate-limiting. The steady-state observed stimulation of HNE cleavage of FRET-peptide substrate by heparin indicates that heparin binding must stimulate the formerly rate-determining deacylation step. Indeed, heparin promotes a 7.8-fold decrease in *k*_2_ value, whereas the *k*_3_ value in the presence of heparin was increased 58-fold (Table 2). These results clearly show that heparin binding accelerates deacylation and slows acylation. Fig. 4B shows that in the steady state, in the presence of heparin, HNE mainly exists as free-enzyme, EH (49.5%) and non-covalently complexed with substrate, EHS (50%) forms; in this condition only 0.5% of the HNE exists in the acylenzyme form (EHS').

Heparin accelerates the hydrolysis of FRET-peptide by NHE; *k*_cat_ value is increased 4.5-fold in the presence of heparin. However, heparin also induces an increase of 2.0-fold in *K*_M_ value; the catalytic efficiency *k*_cat_/*K*_M_ for this substrate in the presence of heparin is 2.3-fold increased. As mentioned above, heparin promotes a marked decrease of *k_2_* (7.8 fold). It also decreases *k_-1_* by 3-fold. Since in the presence of heparin *k_2_* is largely greater than *k_-1_*, while *k_1_* is slightly increased, the catalytic efficiency in the presence of heparin appears mainly limited by *K*_s_, the equilibrium dissociation constant of the non covalent complex ES.

It has been suggested that when an enzyme reaction is diffusion controlled, the conformation and solvation of the substrate and enzyme will determine the rate of substrate association, and hence the specificity [38]. Our results suggested that the notable heparin-induced increase in catalytic efficiency of HNE might be due to a conformational change of the enzyme resulting in a change of the intrinsic kinetic characteristics.

### The Influence of Heparin upon the HNE pH Activity Profile

**Figure 5:**
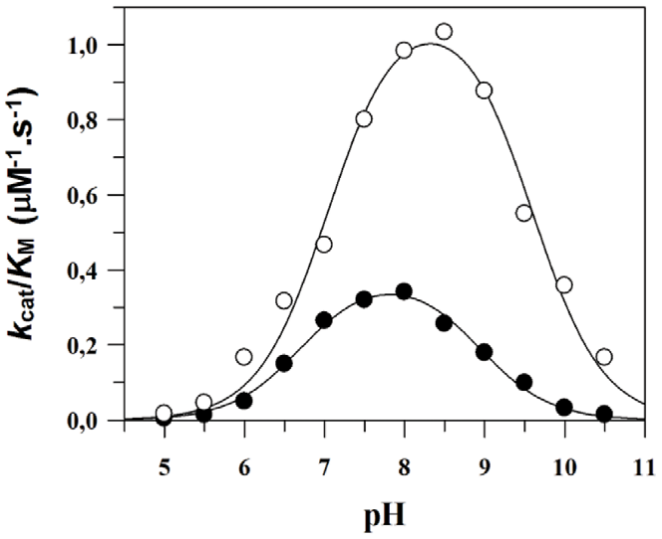
The effect of heparin onHNE pH activity profile. The kinetics of FRET-peptide hydrolysis by HNE were performed as a function of pH in the absence (•) or in the presence (○) of 50 µM heparin at 37°C. The FRET-peptide hydrolysis wasmonitored continuously by the fluorescence of the released product (for experimental details, see the Experimental Procedures section).

The effect of heparin on the pH activity profiles of HNE was analyzed by monitoring the enzyme-catalyzed hydrolysis of the Abz-AMESVMGYFHRSQ-EDDnp substrate. Figure 5 shows that HNE displays bell-shaped pH dependence both in the absence and presence of heparin. When HNE was assayed in the presence of 50 µM heparin a dramatic effect of heparin was observed upon the pH-activity profile. Basically, heparin promoted a general increase of about 2.5-fold in the values of k_cat_/K_m_ observed for FRET-peptide substrate hydrolysis and shifted the HNE pH activity profile about 0.5 units to the right. Table 3 shows that in the presence of heparin the value of p*K*_E1_ was shifted from 6.7 to 7.0, the p*K*_E2_ was increased from 8.9 to 9.6, and the pH_opt_ of HNE was increased from 7.8 to 8.3. These results clearly show that the presence of heparin allows HNE to be active at alkaline pH.

**Table 3:** Effect of heparin upon the ionization constants of prototropic groups from the human neutrophil elastase active site.

Parameters	Control	Heparin
p*K*_1_	6.71±0.05	7.04±0.07
*k*_cat_/*K*_M_(µM^−1^s^−1^)	0.39±0.02	1.11±0.05
p*K*_2_	8.93±0.05	9.60±0.07
pH _optimum_	7.82±0.05	8.32±0.07

The acidic p*K* (p*K*_E1_ of about 6.7 or 7.0) presumably results from deprotonation of His57, while the basic p*K* (p*K*_E2_ of about 8.9 or 9.6) would result from deprotonation of Ile16 [39]. p*K*_E2_ is an apparent p*K*a determined both by the ionization constant of the free α-amino group and by the conformational equilibrium between the active (salt bridge formed) and inactive (salt bridge dissociated) forms of the protease. The salt bridge Ile16-Asp194 is formed when the inactive proenzyme is converted to active enzyme by the proteolytic cleavage of a peptide from the *N*-terminus. Formation of this salt bridge triggers a conformational change in the “activation domain” of trypsin-like enzymes, creating the S1 binding site and oxyanion hole of serine proteases [36], [40].

### Molecular Docking Analysis

**Figure 6:**
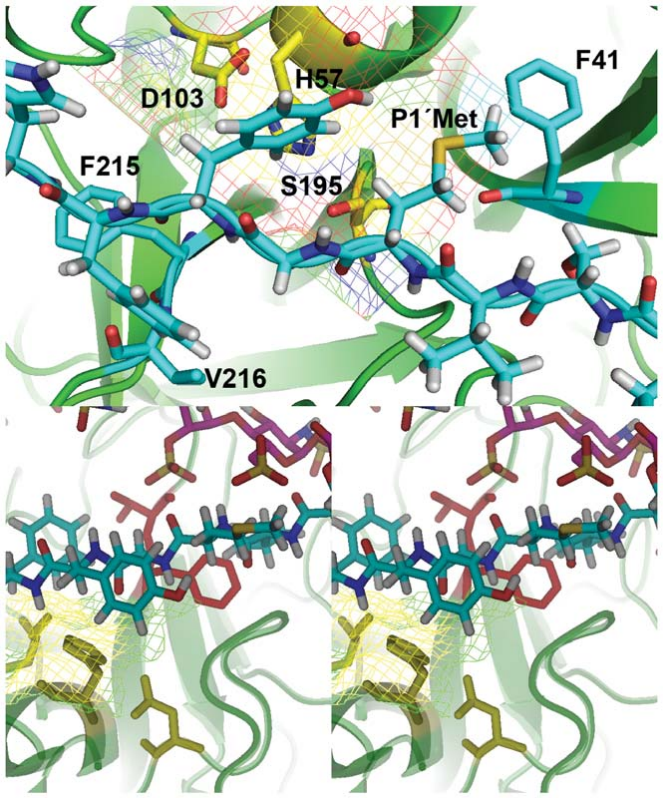
Representation of the complex of both FRET-peptide and heparin with HNE. *A*, Docking of HNE with the substrate AMESVMGYFHRSQ,the secondary structure elements of the HNE are represented by *indigo blue schematics* (*arrows* for extended strands, and *cylinders* for helices).The carbon atoms of the substrate are indicated by a *green sticks*, view of the minimum energy conformation from docking, showing the substrate labeled at P1Val and P1'Met completely engulfed inside the HNE active site cavity: S195, H57 and D103 (dark blue for nitrogen atoms, light yellow for carbon atoms, red for oxygen atoms, grey for hydrogen atoms and dark yellow for sulfur). HNE active site residues are labeled following the numbering of chymotrypsin. *B*, Docking of HNE with heparin,the secondary structure elements of the HNE are represented by *green schematics* (*arrows* for extended strands, and *cylinders* for helices) and the catalytic residues of the HNE are labeled.The carbon atoms of heparin chain are indicated by a *cyan sticks* and its sulfur atoms are indicated by dark yellow. *C*, Enlarged view of the HNE catalytic residues S195, H57 and D103 with substrate labeled at P1Val and P1'Met. *D*, Stereo view of the ternary complex between HNE•Heparin•Substrate, the secondary structure elements of the HNE are represented by *green schematics*. The carbon atoms of the substrate are indicated by a *cyan sticks* and the carbon atoms of heparin are indicated by *magenta sticks*.

Molecular docking of heparin and the FRET-peptide was performed using the crystal structure of the HNE [41], assuming that the minimum energy represents the best model for the peptide binding site. Figures 6A and C show the optimal docking site for the FRET-peptide. It indicates that, as expected, the subsites P1 and P1' of HNE are occupied by Val and Met residues respectively from the FRET-peptide [42]. The carbonyl group of theMet from the substrate is at a 1.21 Å distance from the NH group of the catalytic S195, and the NH group of the Met from the substrate is at a 1.074 Å distance from the CO group of F41 (Table 4). The F41 residue of HNE is in the vicinity of the S1'-S2' substrate binding sites [42]. The CO group of the Tyr residue from the FRET-peptide substrate is within 2.14 Å from the NH group of the catalytic His57 residue (Table 4). In this model, the *2*-*O*-sulfate group from the Idu3 residue of heparin is within 2.4 Å from the carbonyl group of Val216 (Fig. 6B), which is found in subsite P3 of HNE [42]. Heparin interacts with the NH group of Met at S1' (Fig. 6D) and it also perturbs the interaction of Tyr with His 57 at S3' (Table 4). These data indicate that heparin interacts with the substrate binding site that extends from at least subsites S3 to S3' (Fig. 6B). In the presence of heparin, the distance from the Met carbonyl group of the substrate to the NH group of the catalytic S195 was increased from 1.21 to 6.12 Å (Table 4). As previously mentioned, the pre-steady-state analysis shows that heparin was able to decrease 7.8-fold the HNE acylation rate (*k*_2_) of FRET-peptide substrate (Table 2). Taken together, these data suggest that heparin is decreasing the rate of the acylation step of HNE by increasing the distance of the catalytic S195 residue to the carbonyl group of the cleavable amide bond, disturbing the nucleophilic attack of the catalytic serine on the carbonyl group of Met at S1'.Docking analysis of the heparin with free HNE suggests that the heparin is in contact with arginine residues at R36, R75, R76, R80, R177 and R217 (Fig.6B), as previously suggested [4]. On the other hand, in the presence of the substrate, heparin preferentially interacts with HNE at R36, R75, R76, R80, R147 and R149 (Fig. 6D).The theoretical constant of equilibrium obtained for the binding of heparin to HNE is 1.7 nM at 37°C based on the free energy 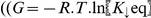). This value is very close to the experimental value of the equilibrium constant (*K_d_* = 3±1 nM), as previously determined for the interaction of heparin with HNE [7]. The docking energy for the FRET-peptide with HNE was −6.6 kcal/mol at 25°C (Fig. 6C). However, in the presence of heparin, the docking energy for the substrate binding to HNE was only −1.3 kcal/mol (Fig. 6D); these data indicate that heparin binding is favoring the release of both the substrate and product from the HNE active site, as previously shown by the data reported in Table 2.

**Table 4:** Molecular Docking Analysis.

Predicted Interactions of the HNE binding site residues with the FRET-peptide substrate			
HNE	Peptide contact	Distance [Å]	Angle [°]
F41 - CO	P1'-Met - NH	1.074	144.8
S195 - NH	P1'-Met - CO	1.21	127.2
H57 - NH	P3' - Tyr - CO	2.14	117.3

Predicted Interactions of the FRET-peptide residues with Heparin			
AMESVMGYFHRSQ	Heparin		
P1'-Met - NH	GlcNS3 - 2-N-SO3-	2.85	121.4
V216 - CO	IduS3 -2-0-SO3-	2.4	114.9

Predicted Interactions of the HNE binding site residues with the FRET-peptide substrate after Heparin docking			
HNE	Peptide contact	Distance [Å]	Angle [°]
F41 - CO	P1'-Met - NH	2.74	144.8
S195 - NH	P1'-Met - CO	6.12	127.2
H57 - NH	P3'- Tyr - CO	7.38	117.3

## Discussion

It had been shown that HNE undergoes inhibition by heparin and related glycosaminoglycans by a tight-binding, electrostatically driven, hyperbolic mixed-type inhibition mechanism with a predominantly competitive character [4]. However, this inhibitory effect upon HNE activity is reversed and even abolished at high concentrations of the glycosaminoglycans [43]. The inhibitory effect of heparin and other polysulfated GAG upon HNE activity seems to be circumstantial rather than consensual and is likely related to both substrate structure [44] and inhibitor concentration [43].

Our results clearly show that heparin can accelerate the rate of TIMP-1 hydrolysis by HNE (Fig. 1). The magnitude of the effect of heparin on the rate of TIMP-1 hydrolysis by HNE is basically the same as the heparin-induced increase of FRET-peptide hydrolysis (Fig. 2). In both cases heparin increases 2.5-fold the hydrolysis of the substrates by HNE. Taken together, these results show that heparin binding is perturbing both HNE substrates hydrolysis in a similar manner. This fact is not an isolated case, since it can be found in the literature that heparin greatly activates a specific and limited proteolytic cleavage of the serpin antithrombin III [ATIII] by HNE [45], [46]. More, heparin and dermatan sulfate accelerated the inactivation rate of the serpin heparin cofactor II [HC II] by neutrophil elastase [47].

Seminal works of Stein et al [21], [34], [48] have shown that the steady-state kinetic parameters *k*_cat_, *K*_M_, and *k*_cat_/*K*_M_ do not give a clear picture of HNE's substrate specificity, because 
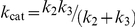
and
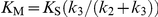
are composite parameters of the intrinsic rate constants. That the determination of the steady-state parameters is not sufficient to describe the kinetic mechanism is particularly obvious in the case of HNE inhibition by heparin.Here, we have shown that HNE exhibits pre-steady-state burst behavior in cleavage of the FRET-peptide substrate related to TIMP-1 sequence (Fig.3). As expected, the acylation rate (*k*_2_) for HNE cleavage of the FRET-peptide Val-Met bond is substantially higher than deacylation (*k*_3_), the product release step. Fig. 3B shows that such behavior reduces the concentration of free enzyme; HNE in steady-state is 71% sequestered in the acyl-enzyme intermediate. The slow rate of deacylation of FRET-peptide suggests that TIMP-1 can be considered a bad substrate for HNE hydrolysis.

The advantage conferred *in vivo* by the rate-limiting deacylation seen with HNE may be not yet apparent. This pre-steady-state behavior reduces the concentration of free enzyme mainly when this potent proteolytic enzyme is secreted to the pericellular environment, thereby lowering the rate of cleavage of substrates at cell surface and extracellular matrix. Similar pre-steady-state behavior has been observed for Kex2 protease and furin, these enzymes are thus thought to accumulate the acyl-enzyme intermediate at the steady state *in vivo* also [49]. It is possible that slow rate of deacylation of TIMP-1 maintains the rate of incorrect cleavage below some toxic threshold, or it may serve as a means of maintaining a reserve pool of protease as a sequestered acyl-enzyme intermediate, since HNE is in excess *in vivo* [1]. However, such a reserve population would only be advantageous if there were some means of mobilizing it to active form of HNE [50]. In agreement with this scenario, we have observed that heparin (Fig. 4) can stimulate *k*_cat_ by increasing the deacylation rate for HNE cleavage of TIMP-1 substrate.

The presence of heparin resulted in a loss of burst kinetics in HNE cleavage of FRET-peptide substrate (Fig.4;) heparin alters the pre-steady-state behavior of HNE not only by accelerating deacylation but also in slowing down acylation rates (Table 2). The pre-steady-state analysis revealed that heparin affects all steps of the reaction: (i) it enhances the ES complex formation, by increasing 2.4-fold *k*_1_ and reducing 3.1-fold *k*_-1_, (ii) it strongly affects the acyl-enzyme accumulation with pronounced decrease in *k*_2_ (7.8-fold), and increase in *k*_3_ (58-fold). The present data suggest that heparin is capable of altering the conformation of the HNE to permit more rapid association with TIMP-1 or FRET-peptide substrate [51]••. However, our data do not exclude the possibility that heparin can stabilize a ternary complex between HNEHepTIMP-1 by interacting with HNE and TIMP-1 simultaneously (Fig. 6 D). It has been shown that heparin interacts with the netrin-like domain at the *N*-terminal region of TIMPs [52].

The effect of heparin towards HNE pH activity profile also suggests allostery as a major aspect of the regulation of HNE (Fig.5).Basically, heparin shifted the HNE pH activity profile about 0.5 units to the right. Table 3 shows that in the presence of heparin the value of p*K*_E1_ was shifted from 6.7 to 7.0, the p*K*_E2_ was increased from 8.9 to 9.6 and the pH_opt_ of HNE was increased from 7.8 to 8.3. These results clearly show that heparin shifts the HNE pH activity profile to the right, allowing HNE to be active at alkaline pH. More, the data also show that the binding of heparin to HNE perturbs the ionization of the reactive imidazolium group of His57 (p*K*_E1_), this residue mediated general basis catalysis of serine proteases [53].

The high affinity of heparin for HNE [13], *K_d_* = 3 nM, is a relevant matter since it results in an increase of HNE activity towards TIMP-1. A previous study presented by Owen and co-workers [9], [10] showed that HLE binds to soluble high *M*_r_ heparan sulfate proteoglycans (HSPG) shed into bronchial secretions from patients with bronchiectasis and into acute human dermal wound fluids [8], [11], retaining its catalytic activity against extracellular matrix substrates. Thus, binding of HNE to HSPG in neutrophil plasma membranes does not significantly compromise its catalytic activities. In this case, the major difference between the soluble and membrane-bound forms of the elastase is its susceptibility to serine proteinase inhibitors, in which the membrane-bound HLE on activated neutrophil are remarkably resistant to inhibition by physiologic inhibitors [8]–[10].

The large amounts of proteoglycans and free chains of GAG present in the extracellular matrix can overcome the slow rate of deacylation of TIMP-1 hydrolysis, thereby increasing the amount of TIMP-1 processed by HNE. Indeed, MMP-9/TIMP-1 imbalance has been observed in sputum of patients with cystic fibrosis [17] and in abdominal aortic aneurysm [19]; where the high level of HNE promotes a large MMP-9 activation either directly or by the proteolysis of TIMP-1, its natural inhibitor. TIMP-1 is essential to protected against blood-brain barrier disruption after ischemic stroke by regulating activities of MMPs [54]. Increased MMP-9 activity is also observed in bone resorption state [20]. Interesting, the major clinical complication of heparin anticoagulant therapy is bleeding, heparin administration can promote hemorrhage complications [55] and osteoporosis by increasing bone resorption [56] as side effects. Heparin was associated with a dose-dependent increase of both intracranial and extracranial bleeding [57]. It has been shown that heparin can produce intracranial hemorrhage dependent of MMP-9 activity [58]. Heparin increases eight-fold the initial rate of proMMP-9 autolytic activation [59]. Here, we are showing for the first time that heparin accelerates 2.5-fold the rate of the TIMP-1 hydrolysis by HNE. Taken together, these data are strongly suggesting that in the presence of heparin a large amount of active MMP-9 is available. These data may have important clinical implications, since the most important adverse effects of heparin are related to excessive activation of MMP-9.
